# Effects of a dietary intervention with conjugated linoleic acid on immunological and metabolic parameters in children and adolescents with allergic asthma – a placebo-controlled pilot trial

**DOI:** 10.1186/s12944-016-0187-6

**Published:** 2016-02-03

**Authors:** Anke Jaudszus, Jochen G. Mainz, Sylvia Pittag, Sabine Dornaus, Christian Dopfer, Alexander Roth, Gerhard Jahreis

**Affiliations:** Present Address: Department of Physiology and Biochemistry of Nutrition, Max Rubner-Institut, Federal Research Institute of Nutrition and Food, Haid-und-Neu-Straße 9, D-76131 Karlsruhe, Germany; Department of Pediatrics, Pediatric Pulmonology, CF-Centre, Jena University Hospital, Kochstraße 2, D-07745 Jena, Germany; Department of Nutritional Physiology, Institute of Nutrition, Friedrich Schiller University Jena, Dornburger Straße 24, D-07743 Jena, Germany

**Keywords:** Asthma, Allergy, Children, Conjugated linoleic acid, Cytokines

## Abstract

**Background:**

Circumstantial evidence suggests that conjugated linoleic acid (CLA) beneficially modulates immune function in allergic subjects. *C*9,*t*11-CLA, naturally occurring in ruminant fats, is suggested to be the effective isomer. In contrast, for the *t*10,*c*12-CLA isomer, which is naturally found only in traces but usually constitutes a relevant part in commercial CLA mixtures, adverse effects have been reported. Aim of this study was to assess putative immunomodulatory effects of highly enriched *c*9,*t*11-CLA in allergic subjects. To our best knowledge, our study is the first in that a CLA preparation was used for such purpose which was free of *t*10,*c*12-CLA.

**Design:**

Twenty-nine asthmatic children and adolescents (age 6–18 y) with diagnosed allergic sensitization against grass pollen, house dust mite, or cat hair/epithelia consumed daily a portion of yoghurt containing either 3 g CLA (75 % *c*9,*t*11-CLA, 87 % purity) or placebo (safflower oil) over a period of 12 weeks. At study start and end, lung function parameters, specific IgE, in vitro allergen-induced cytokine production in peripheral blood mononuclear cells (PBMC), plasma ECP, urinary 8-oxodG as marker of oxidation, fatty acid profiles of erythrocytes, and routine haematological parameters were determined. Prior to blood samplings, 3-days dietary records were requested. Throughout the study, the participants documented daily their peak expiratory flow and kept protocol about their allergy symptoms and usage of demand medication.

**Results:**

In contrast to the CLA group, PBMC-produced IFN-γ and IL-4 increased significantly and by trend, respectively, in the placebo group. Moreover, plasma ECP tended to increase in the placebo group. In the pollen subgroup, FEV1 improved upon both CLA and placebo oil supplementation. In both intervention groups, the *n*-6/*n*-3 PUFA ratio in red blood cells decreased, mainly due to an increase in *n*-3 PUFA. Moreover, 8-oxodG excretion increased in both groups. No changes occurred regarding specific IgE concentrations, allergy symptoms, and volume parameters.

**Conclusion:**

Our results indicate that CLA modestly dampens the inflammatory response on the cellular level. A clinically relevant amelioration of the symptoms could not be proved in atopic manifest patients.

**Trial registration:**

NCT01026506.

## Background

Over the last decades, the incidence of allergic airway diseases such as allergic rhinitis and bronchial asthma has rapidly increased in industrialized countries and currently affects up to 30 % of the population in Germany. In 2008, the direct medical care costs ascribed to asthma accounted for EUR 1.8 billion [1], thus constituting a substantial economic burden. This epidemic trend, which is continuing to rise, has been related to lifestyle changes typical of westernization, such as hygiene, an indoor life, reduced physical activity, and a modified diet. In particular, for the diet hypothesis, epidemiological and cross-sectional evidence has been relatively consistent in suggesting that an unbalanced high intake of *n*-6 polyunsaturated fatty acids (*n*-6 PUFA) such as linoleic acid (LA) may contribute to the incidence of allergic sensitization and asthma (reviewed in [2] and [3]). Research interest increasingly focuses on dietary fats and their detrimental as well as their beneficial roles in the development of diseases. For instance, the consumption of full cream milk and butter from early childhood on was found to be inversely associated with the onset of allergic asthma [4, 5]. Dairy fat, especially that of ruminants being pastured, is the main source of conjugated linoleic acids (CLA), a class of unsaturated fatty acids that comprises several positional and geometrical isomers [6]. We have previously shown that the predominant natural CLA isomer *c*9,*t*11-CLA reduces expression of the chemokine interleukin (IL)-8 in airway epithelial cells [7], inhibits IL-2 and tumor necrosis factor (TNF)-α in T-helper cells [8], and prevents experimentally induced airway inflammation in mice at least in part via a peroxisome proliferator-activated receptor gamma (PPARγ)-dependent mechanism [9]. Based on the in vitro experiments and the data obtained from the animal model, the present pilot study was conducted to investigate the effects of a dietary intervention with CLA on clinical and immunological parameters in children and adolescents with allergic bronchial asthma. Epidemiological studies indicate that patients with chronic diseases often turn to alternative or complementary therapeutic strategies, including dietary supplements [10]. Especially for mild asthmatic children non-pharmacologic interventions could be attractive. Thus, the aims of the study were 1) To provide proof of principle for therapy-supporting usage of CLA in mild asthmatic children, e.g., improvement of asthma symptoms and reduction of inflammatory markers; 2) To assess the clinical efficacy of the CLA treatment, e.g., reduction of demand medication; and 3) To demonstrate whether potential beneficial effects are specific for the CLA treatment (comparison with placebo oil). The novelty of this study is that we used a CLA triacylglycerol preparation which exclusively contained the naturally occurring 9,11-isomers of CLA.

## Methods

### Subjects, study design and study oils

**Fig. 1:**
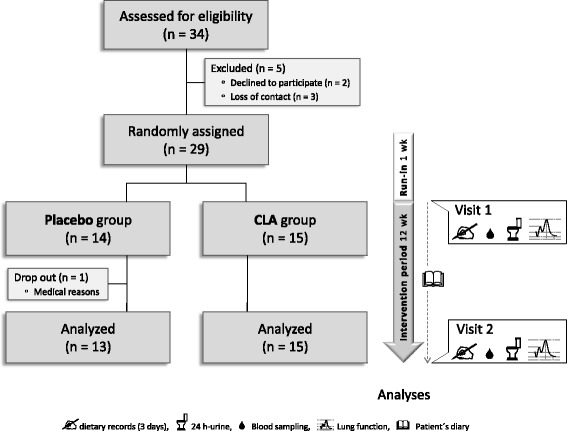
Flow diagram of the study participants recruited and allocated to the study groups, and the design of the study

Twenty-nine children and adolescents (13 girls, 16 boys; age 6–18 years) with diagnosed bronchial asthma were recruited from the Outpatient Clinic for Pediatric Allergology and Pneumology of the Jena University Hospital (Table 1). Eligibility criteria were: allergic sensitization against timothy grass pollen (Phl p1, seasonal allergen exposure), house dust mite, or cat hair/epithelia (Der p1, Fel d1, exposure irrespective of season) to comprise both seasonal and all-year allergy; specific IgE ≥0.7 IU/L (ImmunoCAP, Phadia, Uppsala, Sweden) at screening or within the past 12 month; mild to moderate course of disease defined as clinical stability and mean forced expiratory volume in 1 s (FEV_1_) ≥70 % of predicted at screening; competence regarding the daily documentation of PEF data and of allergy symptoms. Exclusion criteria were: primary and secondary immune deficiency; specific immune therapy within the past 2 years; treatment with anti-IgE-antibodies; regular intake of systemic steroid medication; intolerance against dairy products; obesity (BMI ≥30 kg/m^2^); participation in another study. Before inclusion, written informed consent was obtained from all participants/parents. A total of 28 subjects finished the study (Fig. 1).

**Table 1:** Demographic data and baseline characteristics of the study participants

Parameter	Placebo	CLA
n (female/male)	13 (8/5)	15 (5/10)
Age (y), median (range)	13 (7–18)	13 (6–17)
Age groups, n (% all participants)		
6–12	4 (14)	6 (21)
13–18	9 (32)	9 (32)
BMI (kg/m^2^)^a^, median (range)	19.5 (15.3–29.0^b^)	19.9 (14.7^c^-23.5)
Major allergen, n (% group participants)		
Phl p1	6 (46)	7 (47)
Der p1	4 (31)	5 (33)
Fel d1	3 (23)	3 (20)
FEV_1_ % predicted, median (range)	97 (70–126)	95 (83–122)

^a^Adjusted for clothing

^b^> Percentile 97

^c^< percentile 3 of age- and sex-specific range, according to Kromeyer-Hauschild [54]

The study was designed as a prospective and placebo-controlled pilot trial. After a 1-week run-in period to ascertain the current state of disease, categorization of allergic sensitization, and delivery of the patient’s diaries, of the bins for collecting the 24 h-urine, and of the 3-days dietary record templates, the participants were evenly allocated to one of two study groups with due attention to a balanced distribution regarding age and type of allergy (Fig. 1, Table 1). Participants, clinicians, and lab assistants had no information on the group assignment. Throughout the intervention period of 12 weeks, the participants consumed daily either 3 g of an esterified 9,11-CLA preparation free of *t*10,*c*12-CLA (75 % *c*9,*t*11-CLA, 87 % purity [11]) or 3 g of a placebo oil (Table 2) mixed in 100 g portions of commercial low-fat yoghurt (0.1 % milk fat; Linessa). As placebo oil, safflower oil was chosen to energy balance the *c*9,*t*11-CLA with LA (C18:2*n*-6, Table 2). For sensory reasons, the oils were emulsified in pasteurized skimmed milk (1:2, wt:wt) before stirring into the yoghurt. All processing steps were carried out in inert gas atmosphere and under sterile conditions. The study yoghurt was freshly prepared in 2-week intervals and distributed to the children’s homes on the day of preparation. The parents were instructed to store the yoghurt cups frozen until consumption. During the study, the participants maintained their usual food habits.

**Table 2:** Fatty acid profiles of the study oils [% FAME]. Combined GC-FID and silver-ion HPLC analysis^a^

Fatty acid (common name)	CLA	Placebo oil
C14:0 (myristic acid)	0	0.1
C16:0 (palmitic acid)	0.1	8.2
C18:0 (stearic acid)	0.5	3.4
C18:1*c*9 (oleic acid)	1.8	19.9
C18:1*c*11 (vaccenic acid)	0.3	0
C18:2*n*-6 (linoleic acid)	3.1	64.5
C18:3*n*-3 (alpha-linolenic acid)	0.2	1.0
C20:0 (arachidic acid)	0.1	0.5
C20:1 (eicosenoic acid)	0.5	0.3
C20:5*n*-3 (eicosapentaenoic acid)	0	0
Other	6.8	2.3
∑ CLA	86.6	0
*c*9,*t*11^b^	66.1	
*c*9,*c*11	19.2	
*t*9,*t*11^c^	0.9	
*t*10,*c*12	0.1	

^a^For detailed information, please refer to [11]

^b^Corrected for co-eluting *t*8,*c*10

^c^Corrected for co-eluting *t*8,*t*10

At the beginning and at the end of the study, dietary records on the three preceding days were requested, lung function parameters were assessed by whole body plethysmography and spirometry, and venous blood and 24 h-urine samples were collected for further analyses. Throughout the intervention period, the participants recorded daily their PEF data and kept protocol about their allergy symptoms and demand medication usage. At the end of the study, the patient’s diaries were collected.

The study was registered at ClinicalTrials.gov as NCT01026506, was approved by the Ethics Committee of the Medical Faculty of the Friedrich Schiller University, Jena (approval no. 2079-07/07) and took place at the Department of Nutritional Physiology in Jena.

### Blood sampling, preparation, and measurements

Prior to and after the 12-week intervention period, peripheral blood was taken for immunological outcome measures (in vitro cytokine production of leukocytes, specific IgE, plasma ECP) and metabolic outcome measures (routine haematological parameters, analysis of fatty acid profiles of RBC).

#### In vitro cytokine production

Peripheral blood mononuclear cells (PBMC) were purified from 9 mL heparin blood by density gradient centrifugation. The PBMC interphase was collected, washed three times with PBS and resuspended in VLE-RPMI1640 complete medium/3 % autologous serum. Complete medium contained the following additives: 10 mmol/L HEPES buffer, 2 mmol/L L-alanyl-L-glutamin, 50 μg/mL gentamycin (all Biochrom, Berlin, Germany). 1 × 10^6^ cells/mL were stimulated, according to the individual allergy, either with IgE-binding capable recombinant Phl p1 (Biomay, Vienna, Austria), natural Fel d1 (LTN-FD1-1, Indoor biotechnologies, Warminster, UK), or natural Der p1 (LTN-DP1-1, Indoor biotechnologies) at a final concentration of 2 μg/mL. Corresponding cultures were incubated with the solvent vehicle. After 48 h incubation under standard culture conditions, cells were pelleted, and supernatants were frozen at −80 °C until cytokine analysis with FlowCytomix™ multiplex technology (eBioscience, Frankfurt a.M., Germany). The minimum detectable concentration given by the manufacturer was 4.2 pg/mL for IL-1ß, 16.4 pg/mL for IL-2, 20.8 pg/mL for IL-4, 1.6 pg/mL for IL-5, 1.2 pg/mL for IL-6, 0.5 pg/mL for IL-8, 1.9 pg/mL for IL-10, 1.5 pg/mL for IL-12p70, 1.6 pg/mL for IFN-γ, 3.2 pg/mL for TNF-α, and 2.4 pg/mL for TNF-ß. For some samples, the cytokine concentration assessed on the basis of the mean fluorescence intensity (MFI) was near the test sensitivity limit. This mainly concerned IL-5 and IL-10. In cases where the concentration was below the linear range of the assay with the calibration curve being no longer valid, cytokine concentrations in these samples were assigned the value zero. Thus, to deal with data that were not normally distributed, we calculated the absolute difference between study start and study end values for each subject and compared the study groups regarding the changes in cytokine concentration. MFI values for IL-12p70, TNF-α, and TNF-ß were in most of the samples below the MFI assigned to ‘zero’ and, accordingly, their concentrations not evaluable. Data were evaluated with the FlowCytomix™ Pro 2.2 Software (Bender MedSystems, Vienna, Austria).

#### Plasma ECP

Plasma concentration of ECP was quantified using a commercial ELISA kit (MBL, Nagoya, Japan) with indicated minimum detection limit of 0.125 ng/mL. The assay was performed according to the manufacturer’s instructions. All samples were analyzed in duplicate.

#### Fatty acid analysis

Assessment of the fatty acid distribution was done on the basis of the remaining RBC after isolation of PBMC. Because of their natural numerical ratio of approximately 1000:1, method-related impurity of RBC due to granulocytes was considered marginal.

For gas-chromatographic (GC) fatty acid analysis, a base-catalyzed methylation method was chosen in order to avoid isomerization of conjugated dienes and production of methoxy artefacts [12]. All procedures, GC conditions, used standards, and data evaluation are described in detail elsewhere [13].

#### Routine haematological parameters

Serum cholesterol, triacylglycerol (TAG), creatinine, alanine aminotransferase (ALAT), and aspartate aminotransferase (ASAT) were determined in the routine laboratory of the Department of Clinical Medicine and Laboratory Diagnostics, Friedrich Schiller University of Jena (Head: Dr. Dr. Michael Kiehntopf), according to standard methods of the International Federation of Clinical Chemistry and Laboratory Medicine.

### 24 h-urine sampling, preparation, and measurements

At study start and end, urine was collected during the day before the visits. The participants were instructed to start collecting after the first urination and to keep the morning urine of the following day when the visit took place. 10-mL samples were stored at −80 °C until 8-oxodG analysis. An aliquot of 2 mL was shipped into the routine laboratory of the Department of Clinical Medicine and Laboratory Diagnostics for creatinine measurement.

#### 8-oxodG

Urinary 7,8-dihydro-8-oxo-2’-deoxyguanosine (8-oxodG), a marker of free radical-induced oxidative lesions of nuclear and mitochondrial DNA, was determined by means of HPLC and electrochemical detection as previously described [14]. The renal excretion of creatinine, which is commonly used as reference parameter to adjust for variations in the glomerular filtration rate, increases with the fat-free body mass and was in our study higher in male subjects (mean/median: 8.1/8.6 mmol/L) than in females (6.9/5.7 mmol/L). In order to avoid a possible gender-caused bias, we therefore related the concentration of 8-oxodG excreted during the 24-h period of urine collection to the urine volume and to the body weight (expressed as nmol/24 h/kg).

### Evaluation of 3-days dietary records

In first line, we aimed at assessing the frequency of full cream milk, cheese, butter, and beef consumption, as these foods are the main source of dietary CLA. The per-day uptake of 9,11-CLA was estimated on the basis of the average content of 9,11-CLA in the lipid fraction of these foods. Based on published data on the CLA content of milk fat and beef [15–19], and under the assumption that in 2008 (when the study ran) ca. 98 % of consumed ruminant foods derived from conventional feeding conditions and only 2 % from organic pasture management in Germany, we generated weighed means of the CLA contents, which we used to estimate the CLA uptake from dairies and beef. However, due to limitations of the questionnaire on the one hand and considerable natural variations in the CLA content [20] on the other, it should be emphasized that the herein presented values for CLA uptake represent only approximations.

For reasons of compliance, the second aim of the dietary records was to compare the reported liquid intake with the volume of urine that was collected during the preceding 24-h period. For dietary records, we adapted and modified a template questionnaire, the so-called ‘Freiburger protocol’. In particular, we extended the inquiry by more detailed notes on ruminant fat sources and fat contents. Data were evaluated with Prodi® expert software, version 5.8 (Nutri-Science, Hausach, Germany).

### Defining the pollen season

Data on air-borne grass pollen in and around Jena during the study was kindly provided by the German ‘Pollenflugdienst’ (pollen counting service, Berlin). The start and the end of the grass pollen season (of which the timothy grass pollen season was a subset) were defined as the first and the last 3 consecutive days with ≥5 grains/m^3^/24 h, respectively.

### Statistics

For the statistical analysis, linear mixed models were used with the respective outcomes as dependent variables and study group (CLA *vs.* placebo), study time point (study start *vs.* study end) and an interaction term between the two factors as independent variables. For the pulmonary function and immunological readouts such as in vitro cytokines, the type of allergy (seasonal *vs.* all-year) was additionally included as third fixed effect and the extent of pollination was entered as covariate. Because the subjects underwent examination at two time points, a subject specific random effect was included into all models. Assumptions of these models were judged by inspection of the conditional studentized residuals *vs.* predicted values to assess homoscedasticity of the residuals and QQ-plot of conditional studentized residuals to assess normality of the residuals. In case of the RBC concentrations of *c*9,*c*11-CLA and *t*9,*t*11-CLA, these assumptions were not met and, thus, a Generalized Estimation Equation (GEE) model was applied. When the interaction between study group, study time point and/or type of allergy was significant, a posthoc test was subsequently administered. All calculations were carried out with SAS 9.4 PROC MIXED and PROC GLIMMIX. Unless indicated otherwise, data are given as means ± SD.

## Results

All participants who successfully completed the study reported that they well tolerated the study food. All measured routine haematological parameters were within their normal physiological range. The placebo group had higher TAG values at study start and study end (Table 3), what was in accordance with the higher uptake of fat, especially from ruminant sources, in this group (Table 4). The other parameters were not different between the study groups or the study time points.

**Table 3:** Routine haematological parameters

Parameter	Placebo		CLA		*P* value	
	Start	End	Start	End	gr^a^	gr × ti^b^
Cholesterol (mmol/L)	4.48 ± 0.66	4.39 ± 0.78	4.32 ± 0.76	4.29 ± 0.77	*0.641*	*0.779*
TAG (mmol/L)	1.28 ± 0.69	1.29 ± 0.96	0.90 ± 0.60	0.92 ± 0.55	*0.043*	*0.494*
Creatinine (mmol/L)	66.3 ± 11.6	64.4 ± 9.3	62.3 ± 5.5	63.9 ± 7.9	*0.484*	*0.103*
ASAT (μmol/L × s)	0.49 ± 0.11	0.44 ± 0.09	0.49 ± 0.07	0.48 ± 0.08	*0.562*	*0.186*
ALAT (μmol/L × s)	0.56 ± 0.13	0.55 ± 0.22	0.53 ± 0.10	0.49 ± 0.09	*0.885*	*0.291*

Data are expressed as means ± SD; ^a^study group (placebo/CLA), ^b^study group (placebo/CLA) × study time point (start/end); ALAT – alanine aminotransferase, ASAT – aspartate aminotransferase, TAG – triacylglycerol

**Table 4:** Uptake of macronutrients, ruminant fat, and diet-derived CLA. Per-day values were estimated on the basis of dietary records on the three preceding days prior to blood sampling

Parameter	Placebo		CLA		*P* value	
	Start	End	Start	End	gr^a^	gr × ti^b^
Energy intake (kcal/d)	2087 ± 157	2256 ± 193	1846 ± 199	2031 ± 92	*0.180*	*0.963*
Carbohydrates (g/d)	259.8 ± 22.2	278.4 ± 29.7	238.5 ± 25.4	268.3 ± 17.0	*0.560*	*0.788*
Protein (g/d)	76.3 ± 7.2	81.3 ± 5.7	66.8 ± 7.3	70.9 ± 4.5	*0.141*	*0.936*
Fat (g/d)	79.2 ± 6.7	86.6 ± 7.5	66.9 ± 9.1	71.4 ± 3.3	*0.059*	*0.841*
Fat from ruminant sources (g/d)	21.8 ± 3.8	17.7 ± 2.3	12.2 ± 1.8	12.5 ± 2.4	*0.019*	*0.330*
CLA (mg/d)	131.1 ± 22.9	104.1 ± 12.6	77.4 ± 11.5	81.3 ± 14.7	*0.040*	*0.254*
CLA (mg/100 kcal)	6.1 ± 1.0	4.8 ± 0.5	4.4 ± 0.5	4.0 ± 0.7	*0.087*	*0.474*

Data are expressed as means ± SEM; ^a^ study group (placebo/CLA), ^b^ study group × study time point

### Compliance

**Fig. 2:**
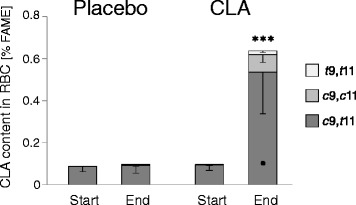
Concentration of 9,11-CLA in RBC. Fatty acid profiles were determined by GC-FID analysis of cellular lipid extracts and are expressed as % of total FAME. Data represent means ± SD. ***Significant increase in all 9,11-CLA isomers compared with placebo and study start (*p* < 0.001)

The fatty acid profile of the RBC serving as middle-term marker tissue is commonly used to reflect food-borne fatty acids. Determining the CLA content of the RBC, we assessed the bioavailability of the supplemented CLA oil on one hand and compliance of the participants with the study diet on the other. The basal level of CLA was approximately 0.1 % of total fatty acids, whereof *c*9,*t*11-CLA, the predominant naturally occurring isomer, accounted for almost 100 % (Fig. 2). After 12 weeks of daily consumption of 3 g 9,11-CLA triacylglycerol, the total increase in CLA within the lipid fraction of RBC was 6-fold (*p* < 0.001). The three 9,11-CLA isomers showed up in RBC lipids at a ratio of 83:13:3 (*c*,*t*:*c*,*c*:*t*,*t*), what corresponded well to their ratio in the oil. There was one sample pair in the CLA group where none of the isomers increased in the RBC lipids, for why low compliance of this participant cannot be ruled out.

### Estimated CLA uptake from the regular diet

We also estimated the CLA uptake from the regular diet on the basis of 3-days dietary records. For the assessment of ruminant fat consumption, butter, milk, dairies, cheese, and beef were factored in. No differences between the study groups and study time points were observed with regard to the intake of total energy and of the macronutrients carbohydrates and protein during the preceding 3 days prior to the blood sampling. However, compared to the CLA group, the children of the placebo group consumed significantly more fat from ruminant sources and, accordingly, more CLA (Table 4). The higher fat intake was reflected by higher TAG levels in the placebo group at both study time points (Table 3). As we instructed the participants to maintain their usual food habits during the study, we assume that the higher fat intake, especially from ruminant sources, has been retained throughout the study. This might have been of relevance for the circulating CLA. However, its content in the RBC lipids of the placebo group, referred to as basal level of CLA, was not different from the CLA group’s basal CLA (Fig. 2).

### Pulmonary function parameters

#### Volume parameters

The vital capacity (VC) is the maximum amount of air that can be expelled from the lungs after a maximum inspiration. The addition of VC with the residual volume (RV) results in the total lung capacity (TLC). The RV is the gas volume that remains in the lung after maximal expiration, thereby preventing alveolar collapse. In obstructive respiratory disorders RV is elevated by air trapping, which causes pulmonary hyper-inflation [21].

For both volume parameters, no changes were observed when comparing study groups, subgroups based on the type of allergy, or time points (Table 5).

**Table 5:** Pulmonary function parameters

Parameter (% predicted)	Placebo		CLA		*P* value		Post-hoc
	Start	End	Start	End	gr × ti × all^a^	ti × all^b^	all^c^
FVC					*0.445*	*0.680*	
Phl p1	97/94	101/96	96/95	97/97			
Der p1/Fel d1	97/99	98/100	90/90	92/90			
RV					*0.761*	*0.402*	
Phl p1	106/109	105/120	114/104	109/97			
Der p1/Fel d1	108/111	123/123	101/95	111/114			
FEV1					*0.793*	*0.041*	
Phl p1	93/91	101/95	95/97	99/96			*0.039*
Der p1/Fel d1	104/112	105/114	97/95	95/95			
MMEF					*0.887*	*0.080*	
Phl p1	69/66	76/70	73/69	77/77			
Der p1/Fel d1	92/93	91/90	87/87	81/84			

Data are expressed as mean/median; ^a^study group (placebo/CLA) × study time point (start/end) × type of allergy (seasonal/all-year); ^b^study time point (start/end) × type of allergy (seasonal/all-year); ^c^seasonal (Phl p1) *vs.* all-year (Der p1/Fel d1)

#### Flow parameters

The forced expiratory volume in 1 s (FEV1) is the volume exhaled during the first second of a forced expiration and is given as %FEV predicted, with references adapted for age, body height and gender. FEV1 is the most indicative parameter to assess bronchial obstruction of the airway system by measuring airflow limitations. It is used to diagnose asthma by proving reversibility of airway obstruction (FEV1 + 12–15 % after inhalation of bronchodilators) or hyperresponsiveness (FEV1 – 12–15 % after challenge by specific or unspecific triggers). The maximum midexpiratory flow (MMEF) is the average expiratory flow between 75 and 25 % of the forced vital capacity (FVC). MMEF is calculated as ½FVC/Δt, whereby Δt is the time required to expire the middle half of the FVC. It reflects small airway patency and is negatively correlated with severity and morbidity of asthma.

Independent of the dietary intervention, FEV1 increased significantly (*p* = 0.041) and MMEF increased by trend (*p* = 0.08) over the study period. Post-hoc testing revealed that this increase was significant in the Phl p1 subgroup (*p* = 0.039) but not in the Der p1/Fel d1 subgroup (Table 5). This outcome indicates an improvement of airflow among the children with seasonal allergy upon both the CLA and the placebo treatment. Of note, the Der p1/Fel d1 subgroup started the trial with airflow values closer to the predicted values than the Phl p1 subgroup.

### Disease monitoring parameters

#### Peak expiratory flow (PEF) and symptom score (patient’s diaries records)

**Fig. 3:**
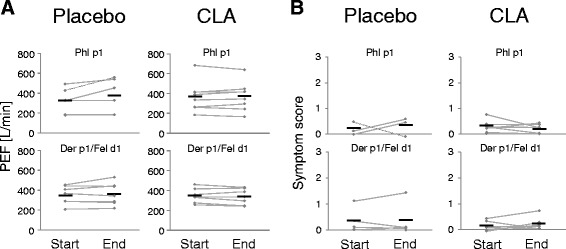
Peak expiratory flow (PEF) and symptom score. The PEF values (**a**) and allergy symptoms (**b**) were self-recorded every day by the participants throughout the study. Of all patient’s diaries, a total of 21 were filled in correctly. Symptom data of a total of 7 participants (5 of the placebo group) were incomplete and therefore not included. Means are defined by crossmarks

PEF is the maximum flow generated during forced expiration following maximal inspiration. For indexing the activity of the disease process, the individual’s best value is taken as reference. At the beginning of the study, the participants received standard peak flow meters for self-monitoring and daily recording their PEF data. PEF values obtained in the morning and the evening of each day were averaged and graphically visualized over the course of the intervention period. The trend in PEF was evaluated according to the slope of the fitted straight line (Fig. 3a).

Allergy symptoms were scored by scaling (sudden) cough, wheezing, and complaints after physical exertion from ‘0’ (none) over ‘1’ (mild) and ‘2’ (moderate) to ‘3’ (severe). Likewise, daily data over the time course were fitted to a trend line (Fig. 3b).

Neither PEF nor the symptom score changed in either of the study groups or subgroups throughout the intervention period. Likewise, there were no changes regarding the usage of demand medication (not shown).

### Immunological parameters

#### Systemic sensitization

Between the start and the end of the study, the mean specific IgE non-significantly increased 1.4-fold in both subgroups of the placebo group, ranging from 0.9- to 2.8-fold (not shown). Accordingly, the CAP class increased in three (all Phl p1) and decreased in one (Fel d1) of 13 participants of the placebo group (each by one point, not shown). In the CLA group, specific IgE remained unchanged in the Der p1/Fel d1 subgroup and increased 1.3-fold in the Phl p1 subgroup, resulting in reclassification of CAP for six participants (two Phl p1 and one Fel d1 increased, and two Phl p1 and one Der p1 decreased, not shown). In one participant of the Phl p1 subgroup of the CLA group, specific IgE increased 10-fold and CAP class changed accordingly from 3 to 6. Interestingly, this was the person from whom the RBC lipid fraction showed no increase in CLA.

#### In vitro cytokine production

**Fig. 4:**
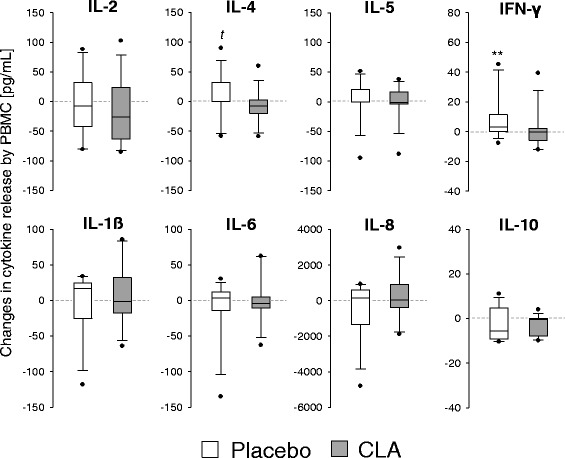
In vitro cytokine production of stimulated PBMC. Box plots depict study start to study end changes in cytokine release. **p < 0.01, ^*t*^ *p* = 0.052

In general and except for IL-8, cytokine release upon allergen stimulation of PBMC was weak. The maximum concentrations detected in supernatants were 140 pg/mL for IL-1ß, 171 pg/mL for IL-2, 100 pg/mL for IL-4, 228 pg/mL for IL-5, 167 pg/mL for IL-6, 5140 pg/mL for IL-8, 25 pg/mL for IL-10, and 62 pg/mL for IFN-γ. For IFN-γ and IL-4, an intervention effect could be described in so far as these cytokines largely remained unchanged in the CLA group and increased or tended to increase in the placebo group (*p* = 0.008 for IFN-γ, and *p* = 0.052 for IL-4, Fig. 4). For all the other cytokines, there were no significant differences, neither within the groups (start *vs.* end; not shown) nor between the groups.

#### Plasma ECP

**Fig. 5:**
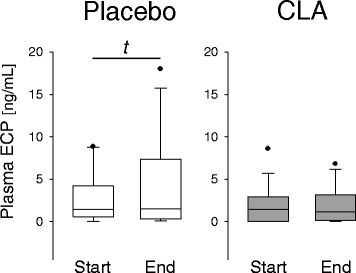
Concentration of plasma ECP

Plasma eosinophilic cationic protein concentrations did not differ between the intervention groups. However, an increase in ECP by trend was observed in the placebo group (*p* = 0.089, 2-tailed student’s *t*-test), whereas ECP remained unchanged in the CLA group (Fig. 5).

### Metabolic parameters

#### *n*-6/*n*-3 PUFA ratio in RBC

**Fig. 6:**
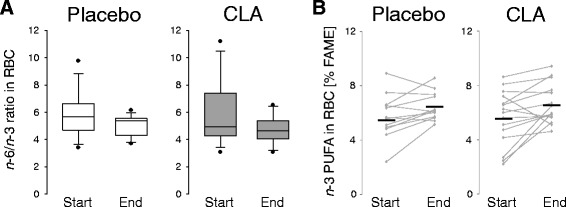
Ratio of *n*-6/*n*-3 PUFA (**a**) and concentration of *n*-3 PUFA (**b**) in RBC. Fatty acid profiles were determined by GC-FID analysis of cellular lipid extracts. **a** The ratio was calculated on the basis of C18-22 PUFA. **b** Means are defined by crossmarks

The *n*-6/*n*-3 PUFA ratio in the RBC lipids was calculated on the basis of all detectable and identifiable PUFA with a chain length of C18 up to C22. Although the main fatty acid in the placebo oil was LA (C18:2*n*-6), the mean *n*-6/*n*-3 PUFA ratio decreased from 5.7/1 to 5.1/1 in the placebo group (Fig. 6a). The mean ratio in the CLA group determined at study start was 5.9/1 and improved to 4.8/1 at study end. Comparing study start with study end, the reduction in the *n*-6/*n*-3 PUFA ratio was significant (*p* = 0.011; Fig. 6a), irrespective of the type of intervention and likely due to a general increase in *n*-3 PUFA in most of the participants (Fig. 6b). Nevertheless, as a ratio of 5/1 is regarded beneficial in asthmatic patients [22], the study end status reflected by the RBC levels was considered as being optimal in both study groups. Moreover and in contrast to the placebo group, in the CLA group the PUFA in sum increased by trend (*p* = 0.08, 2-tailed student’s *t*-test, not shown). However, analysis of dietary records revealed no differences regarding the PUFA intake between the study groups at both study start (CLA: 10 ± 2 g/d; Placebo: 11 ± 1 g/d) and study end (CLA: 11 ± 1 g/d; Placebo: 11 ± 2 g/d).

#### Renal excretion of 8-oxodG

Guanin is the DNA base most vulnerable with regard to oxidation and may easily form 8-oxodG. As a physiological repair mechanism, 8-oxodG is excised and excreted. Hence, urinary 8-oxodG can be regarded as an indicator of oxidative stress, e.g., due to lipid peroxidation [23].

**Fig. 7:**
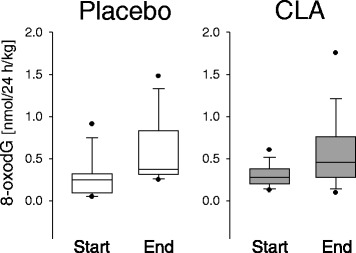
Renal excretion of 8-oxodG

In both diet groups, the urinary 8-oxodG increased significantly during the study (*p* < 0.001; Fig. 7). However, the absolute concentrations as well as the extent of the increase were not different between the two groups.

## Discussion

Almost all studies on the effects of CLA on immune function in humans have thus far been conducted on/in healthy subjects. The first study which addressed the issue that putative effects of CLA could be more pronounced in situations of immune imbalance, such as allergy, was published in 2008 by Turpeinen et al. [24]. This randomized and placebo-controlled trial in subjects with birch pollen allergy yielded encouraging results and indicated modest anti-inflammatory effects of CLA, when consumed daily at a dose of 2 g during the birch pollen season. Although lung function parameters have not been assessed, the aforementioned trial and ours are reasonably comparable in various aspects. Core statement of the two studies is that CLA (with *c*9,*t*11-CLA being the major isomer) might not prevent but certainly dampen the inflammatory response in allergic subjects. For instance, we herein confirm the finding of Turpeinen et al. [24] that consumption of CLA resulted in decreased IFNγ production by stimulated PBMC (Fig. 4). This is of interest since serum IFNγ was associated with accelerated lung function decline in asthmatic patients [25]. Albeit not statistically significant, we showed a dampening effect of CLA also with regard to IL-4 and ECP (Figs. 4 and 5). Both mediators are intrinsically involved in the pathogenesis and manifestation of allergy and asthma [26, 27].

Pertaining to the impact of CLA consumption on human asthma, a prominent study came from MacRedmond et al. [28]. It demonstrated that supplementation of 4.5 g/d CLA as an adjunct to the usual care in overweight mild asthmatics for 12 weeks was associated with improvements in airway hyper-responsiveness. However, there was no difference in FEV1, in ß-agonist reversibility, and in parameters of systemic and airway inflammation between CLA- and placebo-treated groups [28]. The key finding of another trial in asthmatics was that daily ingestion of 4.8 g CLA for 8 weeks did not attenuate airway inflammation or hyperpnea-induced bronchoconstriction [29]. Of note, in all of the above cited studies CLA mixtures have been used which contained *t*10,*c*12-CLA in relevant amounts.

Several former studies have investigated the effect of fatty acid supplementation on asthma, mainly focusing on *n*-6 or *n*-3 fatty acids. The predominant scientific view is that the trend towards increased consumption of *n*-6 fatty acid may have contributed to the rise in the prevalence of allergic diseases [3]. For instance, in one cross-sectional study on young adults, the risk of asthma increased with the plasma concentration of *n*-6 fatty acids [30]. Several other studies found that a high intake of *n*-6 fatty acids and high serum levels of *n*-6 fatty acids were associated with lower FEV1 and increased exhaled nitric monoxide as surrogate for inflammation [31–33]. In contrast, a substantial number of trials reported that a diet high in *n*-3 fatty acids can improve asthma control [34–39]. However, it is not clear whether this benefit persists and, more importantly, a comparable number of studies showed little or no effect for *n*-3 (reviewed in [40, 41], and [3]).

In general, the evidence on the relation between individual fatty acids and lung function is limited. In the present study, the higher intake of *n*-6 fatty acids, mainly LA, in the placebo group did not impair lung function. On the contrary, among the children with seasonal allergy, the flow parameters improved in the placebo group even more clearly than in the CLA group (Table 5).

In our study, consumption of 3 g 9,11-CLA over a period of 12 weeks resulted in an increase in *n*-3 PUFA in the lipid fraction of RBC (Fig. 6b). We observed this phenomenon before in our murine asthma model where the *n*-3 PUFA in RBC rose upon a 9,11-CLA-enriched diet [9]. In a study on prenatal nutritional behavior, in which we analyzed fatty acid profiles in RBC and plasma of mother-newborn pairs, we found that a maternal diet high in milk products and, accordingly, high in vaccenic acid (the precursor of *c*9,*t*11-CLA and of *t*16:1*n*-7 [42]) was positively associated with both fetal *c*9,*t*11-CLA and fetal long chain *n*-3 PUFA [43]. To date, it is not clear how CLA may influence *n*-3 PUFA. It is conceivable that this effect is an indirect, as 9,11-CLA enrichment in our mouse model led to a systemic concentration shift in the fatty acid profile, largely driven by a percental reduction in *n*-6 PUFA [9]. However, there is no plausible explanation for our observation that the *n*-3 PUFA increased in the RBC of the placebo group as well. Of course, we cannot rule out putative effects of other ingredients of the study yoghurt than the supplemented oils. For instance, an interesting finding of Wall et al. is that oral administration of probiotics resulted in increased tissue concentration of *n*-3 PUFA [44]. The yoghurt we used in the study was produced with *Streptococcus thermophilus* and several *Lactobacillus ssp.* as starter cultures. Full particulars were company secret, as we have been informed. Designing the dietary record templates, we focused on the intake of ruminant foods and less on dietary sources of *n*-3 PUFA. Therefore, we have no exact information on the intake of *n*-3 PUFA which could have been considered as confounder. However, the estimated intake of total PUFA was not different between the study groups. Yet, we can only speculate that, likewise, no significant differences have occurred regarding *n*-6 and *n*-3 PUFA intake.

In the present study, a triacylglycerol esterified CLA preparation rich in *c*9,*t*11-CLA and free of *t*10,*c*12-CLA was applied [11]. This characteristic of the oil was of special interest as *t*10,*c*12-CLA, accounting for approximately 50 % of total CLA isomers in commercially available CLA preparations, is associated with lipodystrophic alterations and hepatomegalia in rodents (reviewed in [13]). But also in humans, several studies on safety-assessment revealed unfavorable isomer-specific metabolic effects, namely development of insulin resistance [45] associated with increased oxidative stress and elevated inflammatory biomarkers [46, 47]. Even a first case of acute hepatotoxicity after middle-term ingestion of a preparation containing *t*10,*c*12-CLA was described [48]. During the present study, all measured routine parameters remained within their normal physiological range upon the CLA supplementation and no adverse events were noticed compared with the placebo.

More often than 8-oxodG, urinary 8-iso-PGF2α (an isoprostane generated by free-radical induced lipid oxidation) and/or 15-keto-dihydro-PGF2α (generated from PGF2α via cyclooxygenase reaction) are used for the assessment of endogenous oxidative stress due to lipid peroxidation. It is known that urinary levels of 8-iso-PGF2α and 15-keto-dihydro-PGF2α are increased after CLA supplementation [24, 46, 47], whereby the pro-oxidative effect seems to be primarily exerted by the *t*10,*c*12 isomer of CLA. Other dietary fatty acids such as LA also induce F2-isoprostane formation [49]. Moreover, increased lipid peroxidation-derived DNA damage following high intake of LA was described [50]. Park & Floyd [51] postulated that the formation of 8-oxodG is mediated by lipid peroxidation products. However, in a study conducted by Kuhnt et al. with the aim to evaluate the oxidative stress induced by dietary intake of *trans* fatty acids, no association between urinary 8-iso-PGF2α or 15-keto-dihydro-PGF2α and 8-oxodG was found [14]. In the present study, excretion of 8-oxodG was elevated upon 9,11-CLA supplementation (Fig. 7) what indicates elevated oxidative stress. However, the increase in urinary 8-oxodG was comparable with the placebo group, thus likely not specific for the CLA intervention. Finally, although increased during the study, the study end values for 8-oxodG were still within the normal physiological range [52, 53].

## Conclusions

In summary, the findings of this pilot trial indicate a possible beneficial role of CLA ingestion in asthma on the cellular level of inflammation. But our data provide no evidence for a suggested therapy-supporting value of CLA supplementation in mild asthmatic children at school age. There is consensus that, in early life, a window of opportunity exists for imprinting and maturation of the immune system. It is most likely that the later development of asthma may be influenced also by nutritional factors such as ruminant fatty acids [4]. Maybe, intervention strategies should be focused on this critical time period to be effective in terms of disease prevention.

## Abbreviations

8-oxodG: 7,8-dihydro-8-oxo-2’-deoxyguanosine
Der p1: Dermatophagoides pteronnyssinus allergen 1
ECP: Eosinophilic cationic protein
Fel d1: Felis domesticus allergen 1
GC-FID: Gas chromatography with flame ionization detection
Phl p1: Phleum pratense allergen 1
RBC: Red blood cells
VLE: Very low endotoxin
